# Preparation of a Sustained Release Drug Delivery System for Dexamethasone by a Thermosensitive, In Situ Forming Hydrogel for Use in Differentiation of Dental Pulp

**DOI:** 10.1155/2013/983053

**Published:** 2013-11-27

**Authors:** Elham Khodaverdi, Fatemeh Kheirandish, Farnaz Sadat Mirzazadeh Tekie, Bibi Zahra Khashyarmanesh, Farzin Hadizadeh, Hamideh Moallemzadeh Haghighi

**Affiliations:** ^1^Targeted Drug Delivery Research Center, School of Pharmacy, Mashhad University of Medical Sciences, Mashhad, Iran; ^2^Student Research Committee, School of Pharmacy, Mashhad University of Medical Sciences, Mashhad, Iran; ^3^Medical Nanotechnology Research Centre, Tehran University of Medical Sciences, Tehran, Iran; ^4^Department of Medicinal Chemistry, School of Pharmacy, Tehran University of Medical Sciences, Mashhad, Iran; ^5^Biotechnology Research Center, School of Pharmacy, Mashhad University of Medical Sciences, Mashhad, Iran

## Abstract

In situ forming delivery systems composed of block copolymers are attracting substantial attention due to their ease of use, biocompatibility, and biodegradability. In this study, the thermoresponsive triblock copolymer PLGA-PEG-PLGA was studied as a dexamethasone delivery system. Dexamethasone, a synthetic glucocorticoid, is used clinically to improve inflammation, pain, and the hyperemesis of chemotherapy, and it is applied experimentally as a differentiation factor in tissue engineering. PLGA-PEG-PLGA was synthesised under microwave irradiation for 5 min. The obtained copolymer was characterised to determine its structure and phase transition temperature. An in vitro release study was conducted for various copolymer structures and drug concentrations. The yield of the reaction and HNMR analysis confirmed the appropriateness of the microwave-assisted method for PLGA-PEG-PLGA synthesis. Phase transition temperature was affected by the drug molecule as well as by the copolymer concentration and structure. An in vitro release study demonstrated that release occurs mainly by diffusion and does not depend on the copolymer structure or dexamethasone concentration.

## 1. Introduction

Pharmaceutical science has shown notable advancements in the development of novel drug delivery systems [1] in the form of microparticles [2, 3], nanoparticles [4, 5], and hydrogels [6, 7]. Most of these new carriers are made of polymers and particularly the biodegradable di- and tri block copolymers composed of hydrophobic and hydrophilic blocks, such as hydrophobic polycaprolactone [8, 9], polylactide [10, 11], poly propylene oxide [12], polylactide glycolide [13], and hydrophilic polyethylene glycol [14] and polyethylene oxide [15]. The copolymer structure allows the ready formation of nanomicelles and hydrogels due to physical crosslinking between hydrophobic sections [16]. These copolymers could also show intelligent behaviour with respect to phase transition and drug release profile [17]. Current smart delivery systems are focused on thermo-responsive and in situ gel-forming copolymers because of their ability to assume a gel form abruptly in response to a rise in temperature above the gelation temperature (the temperature at which the copolymer sol turns into a gel). This property makes a copolymer formulation injectable (sol) below body temperature and creates a sustained release system (gel) at body temperature [18–20].

PLGA-PEG-PLGA is a tri-block copolymer that consists of polyethylene glycol (PEG) and two polylactide glycolide (PLGA) blocks; the latter are composed of lactide (LA) and glycolide (GA) monomers. PLGA is a hydrophobic block due to the presence of LA, while PEG is a hydrophilic block. Zentner and coworkers were the first to synthesize this copolymer (Regel) and to demonstrate its thermoresponsiveness and in situ forming properties [21]. The copolymer is biocompatible and biodegradable; therefore, it is a good candidate as a drug delivery system [22].

Different therapeutic cargos such as DNA molecules, peptides (growth hormone [23], venom peptide [24]), proteins (insulin [25]), and other drug molecules (paclitaxol [21], naltrexone hydrochloride [26]) have been loaded into this hydrogel and both in vitro and in vivo investigation have demonstrated the suitability of PLGA-PEG-PLGA hydrogel for therapeutic applications. Various parameters such as PEG molecular weight (Mw), LA to GA ratio (LA : GA), tri-block copolymer Mw, copolymer concentration, and persistence of other molecules in the copolymer formulation (such as drugs and salts) can be manipulated to alter the properties of the system, such as its phase transition temperature, viscosity, swelling, degradation rate, and drug release profile [22, 27–29].

Dexamethasone sodium phosphate (dex), the candidate drug for loading into the PLGA-PEG-PLGA hydrogel in this study, is a synthetic glucocorticoid (GC) that binds to the GC receptors presenting in most mammalian cells. The significant effect of GCs on suppressing immune system responses leads to their extensive use in treating diseases caused by overactivity of the immune system [30, 31]. These drugs have often been prescribed to cancer patients to reduce side effects of chemotherapy such as hyperemesis and toxicity to normal tissue [32, 33]. In addition, GCs exert proapoptotic effects on lymphoid cells [34]. However, Herr and coworkers confirmed that dex treatment induces the resistance of lung and cervical cancer cells to cisplatin, which has raised concerns about the coadministration of chemotherapeutic drugs and dex [35].

One study carried out on bone marrow stem cells indicated that chronic exposure of cells to dex induces their differentiation into osteogenic cells [36]. Ogata et al. showed that expression of the BSP gene in response to dex accounts for the differentiation of stem cells into osteoblasts [37]. Martins and coworkers prepared electrospun scaffolds loaded with dexamethasone as a differentiation factor for oestrogenic induction of bone marrow mesenchymal stem cells [38].

Another interesting use of dex is in dentistry, where this drug is considered as a potential treatment that promotes fibronectin synthesis and suppresses nerve growth factor secretion in tooth pulp [39]. A previous study indicated that dex stimulates differentiation of odontoblast-like cells in human dental pulp cultures, while reducing the proportion of smooth muscle actin-positive cells [40].

In the present study, we assumed that PLGA-PEG-PLGA hydrogel loaded with dex would be an appropriate system for use as a scaffold for tissue engineering and osteogenic induction of mesenchymal stem cells; moreover, it could be used for tooth pulp healing as a sustained release drug delivery system. We studied the effects of drug concentration and copolymer structure (LA : GA) on hydrogel properties. In addition, we investigated the in vitro release of dex from the hydrogel, and the release mechanism and effective parameters of its release profile were explored.

## 2. Materials and Methods

### 2.1. Materials

Glycolide A (GA), D,L-lactide (LA), and stannous 2-ethylhexanoate were acquired from Sigma-Aldrich, USA. Polyethylene glycol (Mw = 1000), acetonitrile (HPLC grade), monobasic sodium phosphate, monobasic potassium phosphate, and sodium hydroxide were purchased from Merck Co., Germany. Dexamethasone sodium phosphate (dex) was provided by Osve Co., Iran.

### 2.2. Synthesis and Purification of PLGA-PEG-PLGA

PLGA-PEG-PLGA was synthesised according to the previously reported method, with minor modifications [26], using a microwave assisted method and a Milestone MicroSYNTH. For this purpose, PEG (MW = 1000) was dried at 120°C under microwave irradiation and vacuum for 10 min. Mixtures of LA : GA with ratios of 3 : 1 or 5 : 1 were combined with stannous 2-ethylhexanoate and added to the PEG reaction. The reaction mixture was again incubated under microwave irradiation at 150°C and 800 w for 5 min.

The resulting copolymer was purified by dissolving the product in 4°C water and precipitating at 80°C. The supernatant contained impurities that do not precipitate at higher temperature and was discarded. The purified PLGA-PEG-PLGA copolymer was then lyophilised.

### 2.3. Hydrogel Preparation

Copolymers were dispersed at concentrations of 17, 23, and 28% w/v into warm phosphate buffer (PBS,  pH = 7.4), and incubated at 4°C overnight with continuous stirring by a magnetic stirrer to dissolve.

Drug loaded hydrogels were prepared by dissolving dex at a concentration of 0.1 and 0.4% w/v in the copolymer solution and transferring the mixture to a water bath (37 ± 0.1°C) until a gel formed.

### 2.4. Triblock Copolymer and Hydrogel Characterisations

#### 2.4.1. ^1^H NMR

^1^H Nuclear magnetic resonance (^1^H NMR) spectroscopy was used to confirm the structure of the copolymers, to estimate the LA : GA ratio, and to calculate the number average molecular weight (Mn) of the PLGA-PEG-PLGA. ^1^H NMR was carried out in CDCl3 using a Bruker AC-80 NMR instrument at 300 MHz and 25°C.

#### 2.4.2. Evaluating the Phase Transition of the Hydrogel

The sol-gel transition temperature was investigated by the inverted test tube method [29]. Briefly, 1 mL of each solution was transferred to as refrigerated bath circulator (P-22, WISD, South Korea), and the temperature was increased at 1°C/min from 0°C up to the point where gel formation occurred and then at the same rate until it precipitated. To confirm gel formation, the vial was inverted every 2 min to evaluate the flow of the formulation. The syringeability of the copolymer sol was evaluated by passing the formulation through a 25 gauge syringe needle.

### 2.5. In Vitro Study of Drug Loaded Hydrogels

After complete formation of the gel (1 mL) (after about 5 min incubation at 37°C), 4 mL of PBS (pH 7.4) was poured above each gel as a release medium. The samples were shaken at  20 ± 2 rpm and incubated at 37°C in a reciprocal water bath (N-BIOTEK NB-304, South Korea). Samples were taken at 0, 8, 12, and every 24 h (until release of the drug was complete) by withdrawing 1 mL of release medium, which was replaced by fresh medium. The samples were analysed by high performance chromatography (HPLC) with isocratic solvent delivery using a Waters (USA) 600 pump system equipped with a C18 column (3.9 × 150 mm Novapak C18). The mobile phase consisted of 70 : 30 acetonitrile : PBS (10 mM,  pH = 3) at a flow rate of 1 mL/min at 25°C. The dex eluted from the column was detected with a UV/VIS detector (Waters 2487 dual absorbance detector) at 242 nm. The volume of each injection was 20 *μ*L and each sample was analysed three times. Data were analysed using AzurV.4.0 software.

### 2.6. Statistics

The results were presented as means ± SDs (*n* = 4). Paired  *t*-tests and one-way ANOVA were used to analyse the results with a significance level of  *P* < 0.05.

## 3. Results

### 3.1. Copolymer Synthesis and Characterisations

Copolymers with LA : GA ratios of 3 (C1) and 5 (C2) were prepared by a ring opening method using microwave irradiation. The yield of this reaction was greater than 85%.

**Figure 1:**
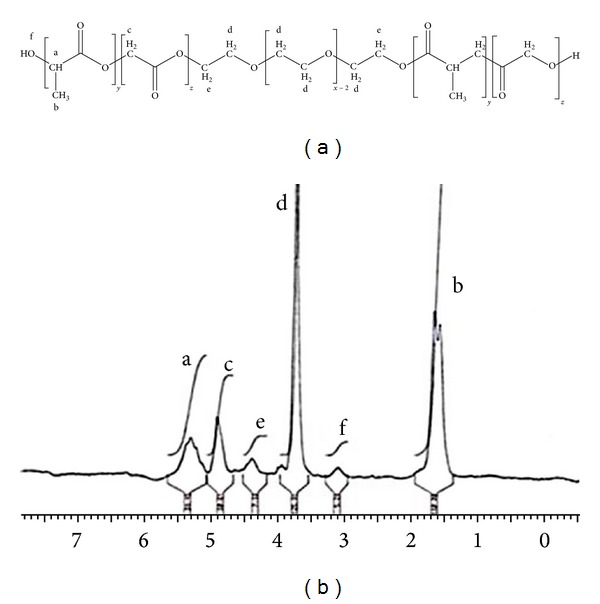
^1^H NMR spectrum of PLGA-PEG-PLGA (LA : GA = 3 : 1); signals a, c, e, d, f, and b are associated with the CH of LA, the CH_2_ of GA, CH_2_ of PEG, another CH_2_ of PEG, and the OH and CH_3_ of LA, respectively.

The ^1^H NMR spectrum of C1 is shown in Figure 1, which shows an acceptable conformity with a previously reported spectrum for PLGA-PEG-PLGA. The signals appeared at 5.2 (a), 4.8 (c), 4.3 (e), 3.5 (d), 3 (f), and 1.5 (b) ppm associated with the CH of LA, the CH_2_ of GA, a CH_2_ of PEG, another CH_2_ of PEG, and the OH and CH_3_ of LA, respectively.

The Mn and LA : GA ratio estimated from obtained spectrums of C1 and C2 are presented in Table 1. These indicate an adequate similarity with theoretical values, confirming the suitability of this synthesis procedure.

### 3.2. Hydrogel Characterisations

The sol shape of the formulations was easily injectable since it passed effortlessly through the fine needle of a syringe. The phase transition temperatures of C1 and C2 were evaluated via the inverted test tube method as the temperature was increased. The sol-gel transition temperatures prior and subsequent to the drug loading are indicated in Table 2. All the formulations assumed the gel condition in less than 20 s after transfer to a 37°C water bath. Decreasing the temperature below the gelling temperature led to a reversal from the gel to the sol condition.

### 3.3. In Vitro Release Study

**Figure 2:**
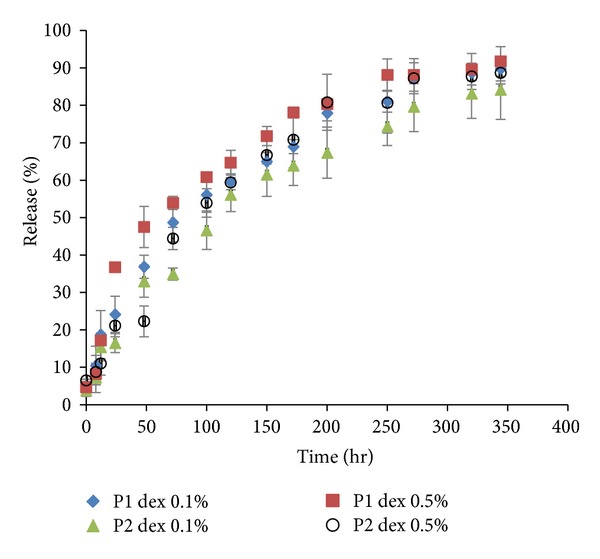
Release profile of dexamethasone (dex) from PLGA-PEG-PLGA hydrogels (mean ± SD).

The C1 and C2 polymers at a concentration of 23% (w/v) were chosen to prepare dex 0.1 and 0.5% (w/v) loaded hydrogels intended for an in vitro release experiment.  Figure 2 illustrates the release profile of dex at various concentrations from the C1 and C2 hydrogels.

The drug release mechanism was investigated by fitting zero order and the Higuchi kinetics models onto the obtained data (Table 3) [41]. Considering the calculated  *R*  square (*R* ^2^), Higuchi kinetics model can navigate the system better, although the  *R* ^2^  of the zero order kinetics model is sufficiently high to articulate that both mechanisms participated in the release of dex. The zero order model indicated that the main release mechanism was polymer erosion. However, when the drug was primarily released by diffusion, the Higuchi model provided a better fit with the data.

## 4. Discussion

A PLGA-PEG-PLGA hydrogel was prepared by a microwave assisted method. As we previously reported, the synthesis of copolymers by microwave irradiation resulted in almost pure and high quality copolymers in excellent yield [9]. The rate of the reaction increases noticeably, which lessens the preparation time from hours to minutes [42]. The conventional synthesis procedure for PLGA-PEG-PLGA takes a long time (about 15 hours), whereas the product is generated in about 5 min using the microwave assisted method.

The ^1^HNMR spectra indicated good accordance with previously reported PLGA-PEG-PLGA spectra, confirming the structure of the obtained copolymers [27]. The obtained copolymers demonstrated the Mn and the LA : GA ratios that were expected according to the amounts of initial materials added to the reaction.

The Mn of the copolymer increased as the LA : GA ratio was raised, since the Mn of each lactide monomer (72 g/mol) is higher than that of each glycolide (58 g/mol). The other parameter that is dependent on the LA : GA ratio is the phase transition temperature. The phase transition occurs due to the specific amphiphilic structure of the PLGA-PEG-PLGA tri-block copolymer, which is composed of hydrophobic PLGA and hydrophilic PEG blocks. Although PLGA is constructed of hydrophilic glycolide and hydrophobic lactide blocks, the amounts of lactide in the synthesised copolymers was 3 and 5 times greater than that of glycolide. As a result, PLGA possessed an overall hydrophobicity.

According to the iceberg theory, when hydrophobic molecules are surrounded by water, this causes a reduction in entropy and the hydrophobic sections aggregate to decrease their exposure to the aqueous phase. Before increasing the temperature and depending on the copolymer concentration and structure, the formulations were in the sol condition, which contained monomers, micelles, and group micelles dispersed in water. As the temperature increased, the hydrogen bonds within PEG and water molecules became weaker. This phenomenon also allows the arrangement of hydrophobic bridges between PLGA segments and finally the material assumes the gel structure. Further elevation of temperature causes phase separation and copolymer precipitation, which also depends on concentration and copolymer structure in a similar way to the sol-gel transition temperature [26, 43].

A LA : GA ratio of 5 led to occurrence of gel formation at a lower temperature due to the prevalence of hydrophobic LA, which causes a higher number of physical crosslinks by hydrophobic interactions. The gel forming temperature also depends on the copolymer concentration. The formulations with higher copolymer concentration could assume the gel shape at lower temperatures due to the higher number of copolymer chain collisions and physical interactions.

Loading of dex reduced the gel forming temperature. Our previous research that examined loading of vitamin  B_12_  and naltrexone hydrochloride showed the same effect, especially with higher concentrations of the drug. We assumed that hydrophilic or soluble salts of drug molecules, by absorbing water molecules themselves, could reduce the water activity and access of PEG segments to the water molecules, thereby accelerating the phase transition at a lower temperature [26, 43].

Evaluating the release profiles indicates that, unlike what was anticipated, neither the copolymer structure nor the drug concentration had any significant influence on the rate of drug release. Since Higuchi model provided a better fit with the obtained data, diffusion appears to be the main mechanism of dex release. The amounts of release approached 100% of the loaded drug before the complete degradation of the hydrogel, so most of drug was released by diffusion. This was not unexpected for dex, given its low Mw and its small size. During diffusion, an increase in the drug concentration led to a higher rate of release [44]; nevertheless, it could also have led to a higher viscosity of the hydrogel (data not shown), as was explained for phase transition. The chance of LA-LA interactions increased due to the salting out mechanism. The water activity also reduces the rate of bulk degradation of the hydrogel, which would affect the rate of solute diffusion by increasing the porosity [44]. The occurrence of these phenomena resulted in an imperceptible increase in the release rate by increases in dex concentration.

Higher LA : GA ratio means more physical crosslinking, more tortuosity, and lower porosity. The size of the hydrogel pores appeared to be sufficiently small for dex to pass through without restraint even at LA : GA 5, and a negligible difference was observed in the rate of drug release between formulations with the same concentration but various copolymer structures. Qiao et al. reported the same findings and indicated that LA : GA ratio has a minor effect on drug release during the diffusion stage. They proposed that the effects of LA : GA depend on the release mechanism and that noticeable effects are seen when the drug is released by hydrogel erosion [22]. Possibly, the comparison between LA : GA 3 and a hydrogel with a much larger ratio (greater than 5) led to this noticeable difference.

## 5. Conclusion

The microwave assisted method is an appropriate technique for the synthesis of PLGA-PEG-PLGA with a high yield of reaction and an acceptable purity. The copolymer structure modulates the phase transition temperature, which is an important factor for developing in situ forming drug delivery systems. However, the structure does not appear to have a substantial effect on the release profile of dex, which is a small molecule, at least for the comparison between LA : GA 3 and 5 carried out in this study.

Loading of a drug can change the physical properties of the system. Drug concentration has a minor effect on dex release rate, although release occurs primarily via diffusion.

## Figures and Tables

**Table 1:** Copolymer composition determined by ^1^H-NMR.

LA : GA		Mn
Theoretical	Practical	
3 : 1	2.9 : 1	3233.18
5 : 1	4.86 : 0	4684

**Table 2:** Gel formation temperature of copolymers.

Copolymer conc. (% w/v)	Drug conc. (%w/v)					
	0		0.1		0.5	
	C1	C2	C1	C2	C1	C2
28	**14.7**	**13.3**	**14.4**	**13**	**14**	**13**
23	**17.4**	**16.2**	**17**	**16**	**16.8**	**15.5**
17	**21.2**	**20**	**21**	**19.5**	**20**	**19**

C1: PLGA-PEG-PLGA with LA : GA= 3 : 1, C2: with LA : GA = 5 : 1; the sol to gel transition temperature (°C) is written in bold.

**Table 3:** Kinetics model of release profile.

Drug conc. (% w/v)	LA : GA	Zero order		Higuchi	
		Slope	*R* ^2^	Slope	*R* ^2^
0.1	3 : 1	0.239	0.893	5.037	0.987
0.5	3 : 1	0.235	0.830	5.068	0.961
0.1	5 : 1	0.232	0.916	4.82	0.984
0.5	5 : 1	0.253	0.892	5.274	0.965
